# Spatially resolved transcriptomics in immersive environments

**DOI:** 10.1186/s42492-021-00098-6

**Published:** 2022-01-10

**Authors:** Denis Bienroth, Hieu T. Nim, Dimitar Garkov, Karsten Klein, Sabrina Jaeger-Honz, Mirana Ramialison, Falk Schreiber

**Affiliations:** 1 Department of Computer and Information Science, University of Konstanz, Konstanz, Germany; 2 Cell Biology, Murdoch Children’s Research Institute, Parkville, Melbourne, VIC Australia; 3 Australian Regenerative Medicine Institute, Monash University, Clayton, Melbourne, VIC Australia; 4 Systems Biology Institute Australia, Clayton, Melbourne, VIC Australia; 5 Faculty of Information Technologies, Monash University, Melbourne, Australia

**Keywords:** Spatially-resolved transcriptomics, Spatial transcriptomics, Virtual reality, Fish tank virtual reality, Head-mounted display, Immersive analytics, Immersive environment

## Abstract

Spatially resolved transcriptomics is an emerging class of high-throughput technologies that enable biologists to systematically investigate the expression of genes along with spatial information. Upon data acquisition, one major hurdle is the subsequent interpretation and visualization of the datasets acquired. To address this challenge, VR-Cardiomicsis presented, which is a novel data visualization system with interactive functionalities designed to help biologists interpret spatially resolved transcriptomic datasets. By implementing the system in two separate immersive environments, fish tank virtual reality (FTVR) and head-mounted display virtual reality (HMD-VR), biologists can interact with the data in novel ways not previously possible, such as visually exploring the gene expression patterns of an organ, and comparing genes based on their 3D expression profiles. Further, a biologist-driven use-case is presented, in which immersive environments facilitate biologists to explore and compare the heart expression profiles of different genes.

**Supplementary Information:**

The online version contains supplementary material available at (10.1186/s42492-021-00098-6).

## Introduction

### Background

The treatment and detection of many genetic diseases rely on the ability to identify gene sets present in diseased tissues or organs, which form candidate genes potentially associated with the disease [1]. Upon defining these gene candidates, the next challenge is to localize them in both time and space because their precise activation in specific cell types is necessary for maintaining normal functions [2]. This daunting task has now become possible owing to the advent of a novel class of ground-breaking technologies, i.e., spatially resolved transcriptomics [3], producing a growing number of high-throughput three-dimensional (3D) datasets. Owing to the lack of appropriate tools used to effectively navigate gene expression patterns, the massive number of transcriptomic data poses challenges in terms of visualization, exploration, and analysis for gaining a better understanding of their underlying mechanisms [4, 5].

It is thus hypothesized that these gaps can be bridged by novel immersive environments (IEs) that enable biologists to effectively visualize the spatial expression patterns of organs in their native 3D context. To investigate this, a framework was designed for a spatial transcriptomics analysis in a single IE, and an interactive visual interface of gene expression values in the human heart was implemented for different IEs. The proposed implementation showcases the potential of an IE for spatial transcriptomics analysis and allows exploring the design choices with the ultimate goal of providing a tool for domain experts.

### Related work

IEs, such as virtual and augmented reality (VR/AR) [6–8] and 3D display walls [9–11], provide the capability to go beyond the conventional computing environment of a desktop setup with a 2D monitor, keyboard, and mouse. They can support stereoscopic 3D data visualization, motion tracking with up to six degrees of freedom, and a wide field of view, and thus broaden the design space for information representation and communication approaches. Such features can significantly enhance the capabilities of a data analysis, with huge potential, particularly for the life and health sciences [12]. However, corresponding solutions need to be carefully designed and tailored toward the use case to facilitate an effective and efficient analysis by human domain experts, avoiding issues stemming from the limitations of both the devices and human perception and cognition [13–15].

Immersive analytics (IA) [16, 17] research investigates the design of data analysis approaches in an IE and exploit the characteristics of such environments, facilitating an analysis. IA further examines how new interactions and display technologies can support analytical reasoning and decision-making. To this end, the impact of the corresponding technological aspects is explored, the integration of analysis methods is considered, and concepts for a smooth and intuitive interaction are developed with the aim of improving the user experience, increasing the efficiency of the analysis process, and supporting the mental state of flow of a user (i.e., the subjective state a person experiences when deeply involved in a task to the point of forgetting all else, including time and fatigue) [18].

However, to create effective and efficient IEs for specific applications and tasks, the large design space of the potential solutions needs to be properly investigated. Design obstacles and potential pitfalls, which may hamper the usability of a system, make it crucial to properly design and evaluate an approach for use in practical applications. For example, although stereoscopic 3D provides a further dimension for data representation and appears to be the natural choice for 3D spatial data, it might increase occlusions, leading to user discomfort, and requiring proper and intuitive techniques allowing the user to easily interact with the data representation and navigate within the environment [19, 20]. Combining spatial and abstract data representations is a further challenge under this setting [21].

The different immersive display technologies fit along the virtual-reality continuum [22, 23], based on their connection to the physical surroundings, i.e., reality. These range from AR systems, such as mobile AR (e.g. [24]), to volumetric and other multi-view displays [25, 26] and mixed reality (MR) systems. They also include systems having a greater extent of world knowledge [22, 27], including HoloLens, large stereoscopic 3D display setups such as CAVE and 3D display walls [9, 11], fish tank VR displays [28] (FTVR) such as zSpace, pure VR systems such as HMD-VR [6, 29], and combinations of systems that include CAVE and FTVR [30], among others. Each type of system has its own characteristics, which might impact its use for different use cases, and therefore might implicate specific limitations or advantages. A comparison of AR and VR systems for an investigation into spatial transcriptomics can elucidate their potential, similar to the example of collective behavior analysis [31]. Naturally, each system type is more nuanced; for example, pure VR devices are most often thought of as head-mounted displays (HMDs). HMD-VR devices can be further differentiated with regard to motion tracking into outside-in and inside-out tracked HMDs. Outside-in-tracked HMDs use external stationary tracking systems such as lighthouses, whereas inside-out tracked HMDs compute the user position and orientation in the environment through cameras and sensors built into the device. Extrapolating from pure VR systems by decreasing the overall immersion and fidelity of control over the mediated world, one arrives at systems such as FTVR. As the name suggests, the virtual world in FTVR is limited to a subset of space around the physical display, extruded along its depth axis. The stereoscopic 3D content is then projected relative to the head-tracked position of the operator. FTVR displays are also stationary, with limited self-motion by the user, and vection [32] causes little simulator sickness. Furthermore, given their form factor, note-taking is also possible, and FTVR displays have recently achieved a generally higher visual fidelity than common HMD-VR devices [33].

Extensive study has been conducted on the electrical, biofluidic, and mechano-physiological simulations of the heart, particularly efforts from the Cardiac Physiome Project consortium [34]. Furthermore, many recent studies have applied VR technologies in the medical education space [35], specifically for cardiovascular anatomy, as exemplified by the Stanford Virtual Heart Project [36]. However, little progress has been made in visualizing the genetics of the heart, partly owing to the lack of high-resolution spatial technologies involved. The emerging spatially resolved transcriptomics field has presented an opportunity to overcome this limitation.

The web application 3D-Cardiomics [37] was designed to visually display gene information in an interactive heart model. It allows the mapping of genetic data with a resolution of 18 heart segments, while providing additional gene expression values sorted according to the distributional similarity to a selected gene. Further related studies have involved 3D atlantes such as the interactive 3D atlas representing a developing mouse heart by de Boer et al. [38] and the Human Brainnetome Atlas [39]. In contrast to several frequently applied web-based approaches, focus is given to a 3D atlas in an IE. Challenges to the creation of IA solutions are discussed for the case of brain activity data used by Jaeger et al. [40]. In addition, Southworth et al. discussed the use of extended reality technologies for cardiology [41], Sardeghi et al. presented a concept for the use of VR in preoperative planning, Brun et al. [42] presented a feasibility study for preoperative diagnostics, and Moro et al. [43] discussed the effectiveness of using VR and AR in the area of medical anatomy.

The increasing possibilities of IEs are allowing an increasing number of three-dimensional models to be used in data mapping. A 3D atlas allows for an improved understanding of the three-dimensional data while enhancing the intuitive understanding of the data mapped onto it.

### Contributions of this paper

Based on the overall idea of 3D-Cardiomics, related data, user feedback, and discussions with domain experts from both cardiovascular biology and immersive analytics, the VR-Cardiomics and methodologies discussed in this paper were designed. The aim is to support users benefiting from model exploration and interpretation in immersive environments. For example, VR allows the intuitive integration of a variety of additional comparison functions.

Herein, VR-Cardiomics, an immersive gene expression atlas of an adult murine heart generated from RNA sequencing of 18 anatomical sections, is presented. In addition, a novel visualization interface is implemented that facilitates interactive gene expression navigation, a synexpression analysis, and differential gene expression patterns across sections, utilizing an interactive VR environment. How different IEs (e. g. FTVR, HMD-VR) can be used and how the proposed platform enables the identification of spatially restricted genes at an unprecedented resolution are demonstrated.

The remainder of this paper is structured as follows: The following section describes the methods used, which are divided into the design, prototype implementation of an HMD-VR and FTVR, and the interaction between the HMD-VR and FTVR presenting adaptions of VR-Cardiomics for FTVR (zSpace) and head-mounted displays (Oculus). In addition, a section detailing the interaction is provided to highlight the device-based differences in the application. The Results section on page 21 presents the target systems and the functionalities of VR-Cardiomics, as well as the use cases, which are provided on page 25. The section also describes examples of relevant research questions and how VR-Cardiomics might be capable of answering these questions. The Discussion section on page 29 then discusses the advantages and problems of the new approach. Finally, some concluding remarks and an outlook regarding future research are provided.

## Methods

### Design

VR-Cardiomics is a stereoscopic 3D application that has been designed for different IEs based on use-case-specific requirements captured from intensive discussions between domain experts and immersive analytics developers. It has been developed as a standalone application for IE, with a focus on the adaptability to different immersive platforms. The heart model, spatial gene expression data, and visual elements were based on the authors’ previous study on a 3D-Cardiomics web application [37]. This has been further developed into VR-Cardiomics with additional IE-specific functionalities.

VR-Cardiomics was developed for either FTVR or HMD-VR. Because the FTVR version resembles an external desktop, this strength has been exploited, and the application is therefore designed for environments similar to a web application. Nevertheless, the three-dimensional feature of the FTVR was added to the respective heart models as the focus of the application. User interfaces and a menu navigation are continuously present on the screen. The size of the heart model in FTVR is perceived to be smaller than that in the HMD-VR version. In addition, the limited screen size leads to a general reduced overview of the environment and model.

**Fig. 1:**
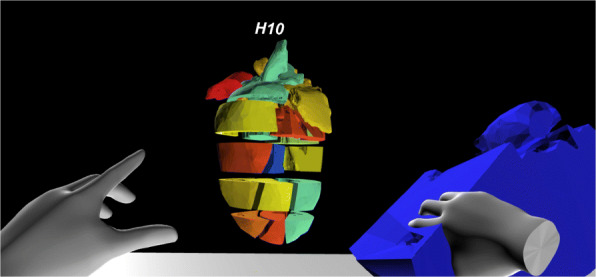
HMD-VR environment with the interaction of a single slice of the heart model

By contrast, the HMD-VR version was implemented in a more explorative manner. Because an HMD is used, the user is provided with an entire 360^∘^ virtual environment. Based on the limited screen size of the FTVR compared to the virtual space of an HMD-VR, the FTVR is fixed for a side-by-side comparison of two models simultaneously, whereas HMD-VR allows multiple models to be simultaneously compared. The benefit of a larger area also results in a larger perceived heart model. An example of a heart model in VR-Cardiomics is given in Fig. 1, the gene expression calculations of which were obtained from bulk RNA-sequencing based on a previous study by Mohenska et al. [37]. RNA sequencing was conducted for each of the 18 areas of the heart, and the expression levels obtained were mapped onto a 3D heart model in Unity using a linear interpolation of a color gradient, from blue for low expression levels to red for high expression levels. Alternatively, for color vision deficiencies, a two-color gradient from blue to yellow was chosen. The menu panel in VR can either be used as a 3D canvas or as a portable touchpad.

The current HMD-VR menu navigation has not been tested through an adequate user study, and design alternatives still need to be explored. To exploit their full potential, detailed user studies and evaluations are necessary for both prototypes.

### Prototype implementation HMD-VR

For the HMD-VR environment, Oculus Rift S and Oculus Quest 2 in link mode were used as inside-out tracked HMDs, applying a state-of-art, generic implementation. Thus, the environment can be adopted for other HMDs. The *Oculus Integration SDK version 28.0* was implemented in Unity to include basic functionalities.

**Fig. 2:**
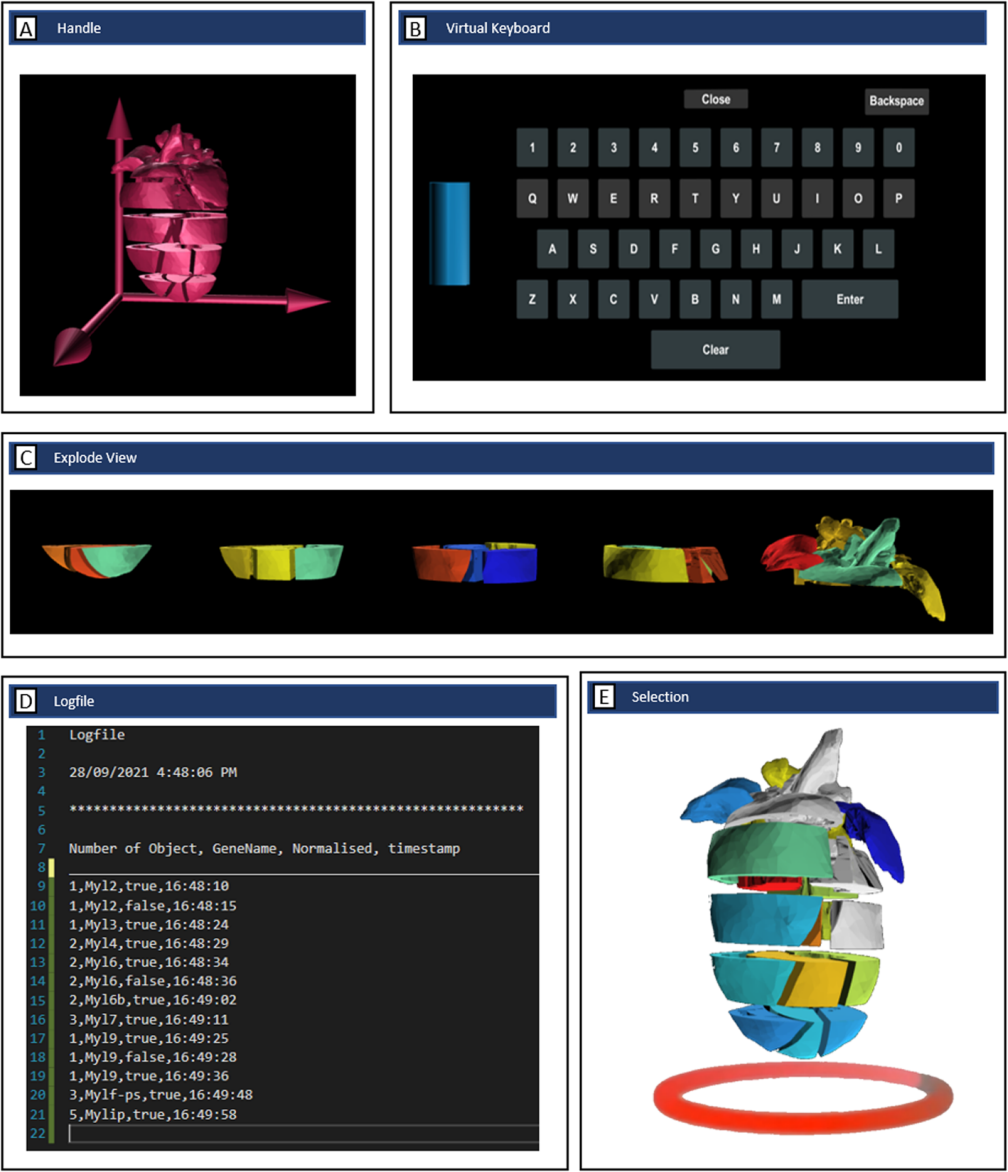
VR-Cardiomics features. A) Handle for rotating the heart model of the HMD-VR prototype. B) Virtual keyboard with a handle for text input. C) Example of expanded features of the heart model to provide a better overview of the heart slices in both environments. D) Backup file created during runtime to keep track of all selected gene expressions, which model the genes were expressed on, information regarding normal or absolute views, and a timestamp. E) Example selection of heart model

In the following short description of the interaction, the 18 slices of the heart model can be moved, rotated, and enlarged individually (Fig. 1). A handle that can be gripped and rotated allows the entire model to be moved as a single unit (Fig. 2 A). Using the menu button, the entire model will be expanded horizontally in front of the user (Fig. 2 C), which allows the internal heart sections to be viewed while providing an overview of all pieces at the same time.

Several input and output features have been elaborated to support the user while applying the HMD-VR, such as a backup file created as a background process for each session (Fig. 2 D). A chronological data list is generated, which stores all selected genes that have been visualized onto the model and the compared expression patterns. In addition, export functions for the tabular results, such as the *similar genes* table, have been introduced to generate a text file in CSV format. In addition, the application has various screenshot and recorder functions that can inter alia capture the user’s current field of view, either as a screenshot in a PNG/JPG format or as a video recording in mp4 format, as well as simultaneous images from four different angles to the heart model. Therefore, all other visual components in the environment are disabled for a duration of two frames (0.03 s) while a screenshot is taken to avoid blocking the view of the cameras to the heart model.

### Prototype implementation FTVR

As a comparative model for FTVR, a zSpace *All-in-One 200 24-GL* monitor was used, which is operated through the computing power of an external PC. To enable core interactions with objects and UI elements within Unity, the *zSpace core SDK (version 6.0.0.11)* was integrated, which had to be adapted to the present needs owing to the EOL of the *zSpace 200 series* in 2018.

The FTVR zSpace resembles a traditional 2D computer monitor; thus, the use of zSpace will seem familiar to the user. Both applications are based on the same functionalities and features. Differences arise only from a technical point of view. zSpace requires specific integration modules integrated in the SDK mentioned above to allow interaction with objects and UI elements. All UI elements, as well as all interactive objects from the VR-Cardiomics implementation for the HMD-VR, had to be adapted to zSpace accordingly. All comparison functions from the previously described VR applications, such as the *combined view*, *heatmap comparison*, and *group selection*, were adopted for a side-by-side comparison of the two models for zSpace.

Several adaptations were made based on this device. For example, a handle (Fig. 2 A) to move the entire heart model was substituted by removing the single-piece interaction of the heart slices. This implementation was omitted because it did not improve the handling, and had a negative impact on the intuitiveness of the application. The selection of the individual heart slices for the *group selection* was transferred to the stylus instead, allowing the environment to be completely controlled by the stylus pen with the exception of a keyboard input for a gene search.

### Interaction: HMD-VR

**Fig. 3:**
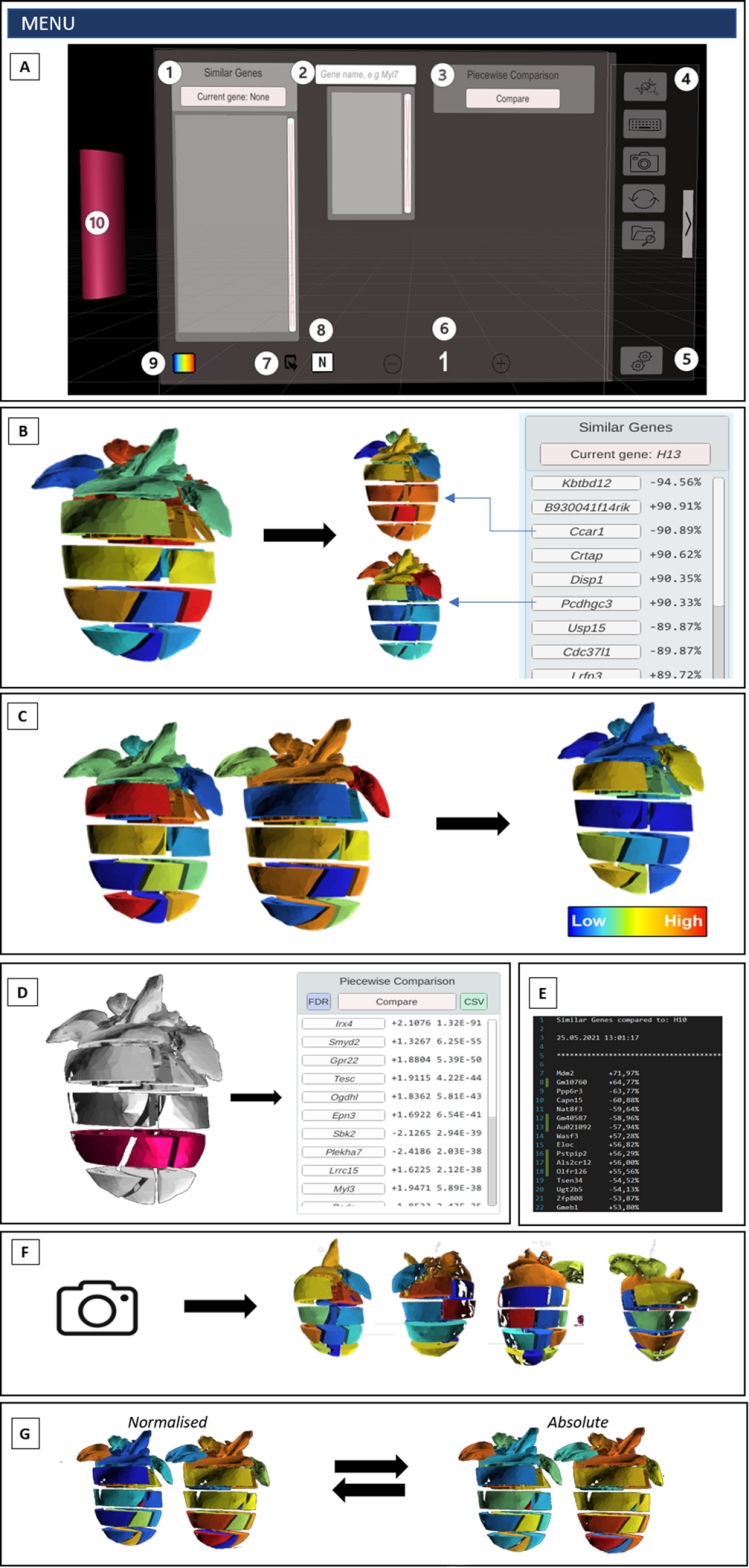
**Features and functions of VR-Cardiomics.** A) VR-Cardiomics menu panel (HMD-VR), with all features of VR-Cardiomics, which are also valid for an FTVR with the exception of features 4,5,6, and 10. 1) A sorted list of genes with similar expression patterns as the selected gene. 2) Search bar with complete auto features. 3) *Piecewise comparison/group selection* feature. 4) Sidebar panel, functions from top to bottom: switch to current view of menu panel, toggle virtual keyboard, screenshot function, reset, and dataset selection. 5) Settings menu. 6) Add or remove 3D objects to or from the environment. 7) Export *Similar Genes* list. 8) Toggle between absolute or normalized expression pattern visualization. 9) Color spectra for expression patterns. 10) Handle used to move the menu panel B) Visualization of a gene expression pattern in the heart model with an example of a resulting similar gene list. C) Side-by-side comparison of two different gene expression patterns (left) and *heatmap comparison* of two genes within one model based on the differences in the gene expression values in each section (right). D) *Piecewise comparison/group selection* to analyze the gene expression based on the regions within the model. E) Similar gene list exported as text files. F) Example of a screenshot from four different angles of the heart model. G) Comparison of visualization of normalized and absolute values

Using HMD-VRs, the user can be immersed in an entire 3D virtual world, providing the opportunity to interact with objects and the environment in a unique way. VR-Cardiomics attempts to exploit this unique feature to its full potential by providing numerous interaction possibilities between the user and the visualized expression patterns on the 3D models. The HMD-VR application was developed using Oculus Rift S and Oculus Quest 2 in link mode. Both devices use Oculus touch controllers. Each controller has three physical and two trigger buttons, supplemented with a joystick for navigation within the environment. Finger positions are recorded using sensors to allow gesture control, such as grabbing or pointing. The main component of VR-Cardiomics is its menu panel, which can be used as a portable touchpad or a fixed menu canvas that can be used through a point-and-click approach. Both options are always present and can be used in a way that best suits the user. Point-and-click refers to how the user points the controller in the direction of the button on the menu panel and confirms the selection by pressing a controller button. Otherwise, the menu can be moved by grabbing the attached pink handle of the menu (Fig. 3 10). The touch controller recognizes whether the user is pointing in a certain direction with an index finger, imitating the gesture in VR. The user can therefore use the index finger to point and touch a button on the menu panel, receiving short vibration feedback from the controller to confirm the selection. A certain expression value from the dataset was selected using the menu panel. Therefore, a virtual keyboard was designed to allow a text input in the search bar of the menu panel. Touching the search bar (Fig. 3 2) or the keyboard button (Fig. 3 4) allows the keyboard to appear in front of the user. The keyboard is used similarly to the menu panel by being moved using the attached handle and allowing an interaction through a point-and-click/touch motion. According to the current input, the dataset is searched for matching gene names and will show the results in a list below the search box. The user can scroll through this list and select from the results of the gene expression visualized on the model.

Because a virtual environment is suitable to allow more models to be used at the same time, a function to select one of the current models was implemented. By pressing the button of the touch controller, a red illuminating circle appears below the first model. Pressing the button again will move this circle to the next model. The current selection is confirmed by pressing an additional button on the touch controller. For all further single-object operations, such as selecting a gene from the dataset, switching between an absolute or normalized expression visualization, or using the expanded feature, the selected object will be applied.

In addition to the interaction with the UI elements of the application, the user can also interact with the 3D heart in several ways. Each model added has an additional handle, as presented in Fig. 2, A. This handle is an extension of the 3D model and is used to move and rotate the model. In addition, the user can interact with each of the 18 pieces of the model individually. Therefore, the piece can be grabbed by bringing the controller to the piece and pressing both trigger buttons on the touch controller. To resize the piece, the user can grab it with both controllers simultaneously and move the controllers away from each other, resulting in an enlargement of the piece. The same is true for the opposite movement used to make the piece smaller again.

Because a direct interaction with an object is a feature unique to an HMD-VR, the authors tried using it for the functions of VR-Cardiomics to allow an intuitive and exploratory interaction with the data. Therefore, the above-mentioned heatmap comparison, used to compare all pieces of the two heart models concurrently, is implemented in a drag-and-drop-like manner. Thus, the handle of one model is grabbed and overlapped with the second model to be compared with. Releasing the handle at this position results in an automated comparison of both models, resulting in one model presenting the intensity of the differences of each piece in a heatmap-like manner.

The group selection also benefits from these interactions because pieces for both groups are simply selected and deselected by touching them and confirming the selection by pressing a button on the touch controller. The corresponding piece will be visually highlighted, and a short vibration feedback will be received to confirm the selection.

The avatar movement within the environment is applied by using the joysticks of the touch controller; therefore, the environment can also be used while seated. The controller of the dominant hand moves the avatar, whereas the other controller rotates the avatar around the axis.

### Interaction: FTVR

**Fig. 4:**
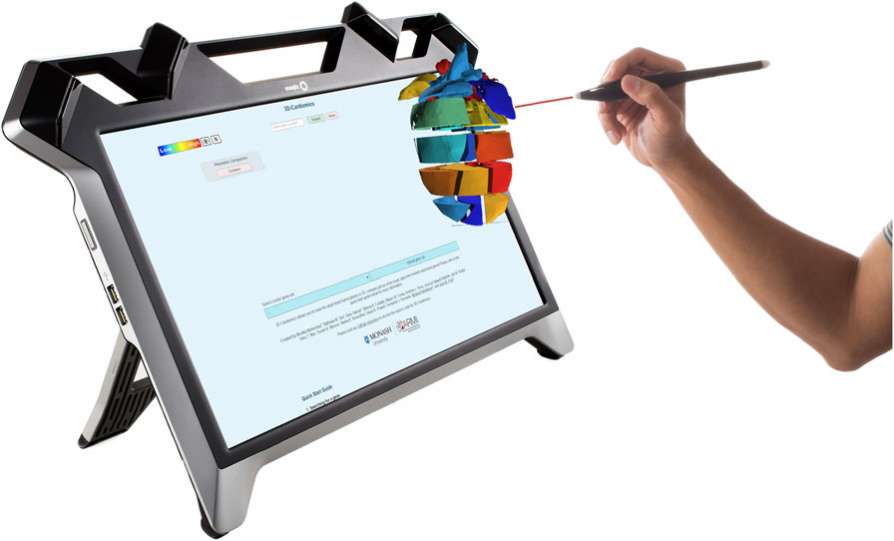
Exemplary representation of 3D-Cardiomics zSpace (FTVR)

Interaction with zSpace is mainly accomplished using the zSpace stylus pen. Alternatively, input devices such as keyboards or computer mice can be used. The user is required to wear polarization glasses with tracking markers to simulate the user’s perception of a 3D object (Fig. 4). The movement of the user’s head is calculated according to the tracking markers, and the 3D object rotation is adapted to the movement of the head accordingly.

With the stylus pen, the 3D object can be moved, rotated, or selected (Fig. 4). Likewise, it can be used to control UI elements of the inter alia menu functions. This pen features three physical buttons and is traced by the trackers of the zSpace desktop. Interactions with the menu elements is achieved by pointing at them and pressing the physical buttons to confirm the selection. To provide the users with a sense of where they are pointing at, a visual laser beam is displayed from the end of the pen to the intersection with an object. Because the stylus pen only offers a total of three buttons and gesture controls are not supported, certain functions have to be implemented as buttons in the menu.

The menu is therefore always present on the screen surrounding the 3D heart, which is used for data interaction. Owing to the technical differences of the device, an implementation based on the web application was more feasible for zSpace. However, most of the features are still implemented, and thus the application is also comparable to a VR application.

This means that the selection of a gene expression from the dataset is conducted using the stylus pen instead, as mentioned above. The user has to click the search bar with either a stylus pen or a computer mouse. Text input is achieved using a keyboard, resulting in scrollable suggestions from which the user can select the expression pattern to be used. Because zSpace is based on a side-by-side comparison of two models at once, the selection of the object is automated. The second heart object can be used once the expression pattern for the first heart is selected. Two additional buttons, one to enable a second 3D heart for comparison (*Combined View*), and one to enable the heatmap comparison of all 18 pieces simultaneously (*Heatmap*), will appear next to the search bar. Both models are interactive and can be moved and rotated by pointing at them and pressing one of the physical buttons of the pen. The model reacts to the movement of the pen as long as the button is pressed. The 3D models are bound to either the search bar or a list of similar gene results. Selecting a button of similar genes leads to a colorization of one of the models according to the expression pattern, whereas the other remains with the previous expression pattern of the search bar. This heart object can be changed using the search bar for a new expression pattern from the dataset.

Pressing the *heatmap* button will result in a comparison of both of the current expression patterns for each single piece projected onto one of the models, similar to the HMD-VR application mentioned above. In addition, the *group selection* feature is similar to that of the HMD-VR version. The selection of individual heart pieces for the *group selection* is implemented using the stylus pen, allowing the user to point at a piece and confirming the selection by pressing the button on the pen, which results in a color-based highlighting of the selected piece.

## Results

### Target systems and functionality

#### Target systems

The presented VR-Cardiomics application was implemented for two different IE concepts, i.e., for (inside-out) HMD-VR and FTVR displays to capture the versatility of each IE depending on the current setting–tasks and users. For instance, the FTVR implementation naturally suits a co-located collaboration, although not unlimited, whereas the HMD-VR implementation more easily allows collaboration across locations. Correspondingly, the discovery of spatially resolved gene expression patterns across tissues pertains to the ability to quickly query and explore the results. Therefore, the expectation is for the FTVR to fit well when the number of target queries is high, whereas in HMD-VR, greater focus can be given to spatial exploration.

Both application environments were created using C# and *Unity3D* [44], a game development engine that has found wide community acceptance and has been commonly adopted for the creation of various virtual environments. The Unity versions used for development are *2019.4.19f1* for HMD-VR and *2018.23.f1* for FTVR. Owing to the computing power required, a VR-capable computer is required. The system was implemented and tested on a workstation computer with an *Intel Core i7-10750H CPU*, a *NVIDIA GeForce RTX 2060* graphics card, and 16GB of RAM. During the tests, the authors observed an average frame rate of 72.04 FPS in an ideal state (six heart objects with a total of 742,716 triangles). This is satisfactory for an interactive analysis and shows sufficient potential for further development and extension.

#### Functionality

A graphical overview, which is based on the HMD-VR menu panel, of the main functionalities of VR-Cardiomics is shown in Fig. 3. VR-Cardiomics is a prototype framework application that supports the visualization of expression patterns combined with three-dimensional segmented objects such as the heart model and different ways to explore the heart object and its attached data in IEs. Gene expression data can be visually analyzed for abnormalities or anomalies, which can reveal peculiarities or correlations that might be disregarded using a common data analysis.

VR-Cardiomics provides an interactive user interface containing all included functions for gene analysis as well as various options for data export and customization options, as shown in Fig. 3. The nature of the FTVR and its limitations to a side-by-side comparison led to a simplified version of the menu for the FTVR. Therefore, the sidebar panel including the settings, the option to add or remove objects, and the handle of the menu panel are omitted in the FTVR (Fig. 3 (4,5,6, and 10)).

The gene to be examined was entered into the search bar (Fig. 3 A,1) using either a conventional keyboard for zSpace or a virtual keyboard generated within the environment for the HMD-VR. Gene selection leads to a coloring of the 3D heart model using the associated gene expression values. This visual representation embodies the normalized gene distribution in each of the 18 heart slices, which can also be displayed in absolute values (Fig. 3 G).

For each gene pattern, a list of genes with similar expression patterns was generated in tabular form (Fig. 3 B). To simplify the interpretation of these results, all genes are listed as buttons to directly visualize them onto the model. The currently displayed selection of similar gene structures can be exported locally as text files in a TSV-like manner (Fig. 3 A,7). In addition, a series of images of the heart model can be generated using the camera icon in the sidebar panel (Fig. 3 A,4) leading to a series of images from four different angles to the heart object (Fig. 3 F).

Several functions have been integrated to achieve an easier comparison of the multiple expression patterns of different genes. One major implementation allowing a visual data comparison in IE is enabled using additional fully intractable and independent heart models (Fig. 3 A,6). An exemplary representation of such a comparison is shown in Fig. 3 C on the left. Using the FTVR application, the second heart model will be colored based on the next gene expression value chosen from the *similar genes* list, which allows the user to interact with two independent heart models with different gene expression patterns at the same time. In the HMD-VR, multiple objects can be placed within the environment, and gene expression patterns can be visualized independently. This function is particularly useful when examining individual areas but shows weaknesses when comparing multiple expression patterns because 18 sections of each heart have to be compared simultaneously. Consequently, a second comparison function was devised, along with a *heatmap comparison* (Fig. 3 C, right), which differentiates the individual expressions of each segment for the two selected genes, and colors them using a color gradient depending on the absolute or normalized differences. The sections of the heart with high absolute/normalized distances are shown in red, whereas shorter distances are shown in blue.

In addition, the *group selection* introduced in 3D-Cardiomics [37] was implemented (Fig. 3 D). This feature can be used for a piecewise comparison of the 18 fragments within a single model.

**Fig. 5:**
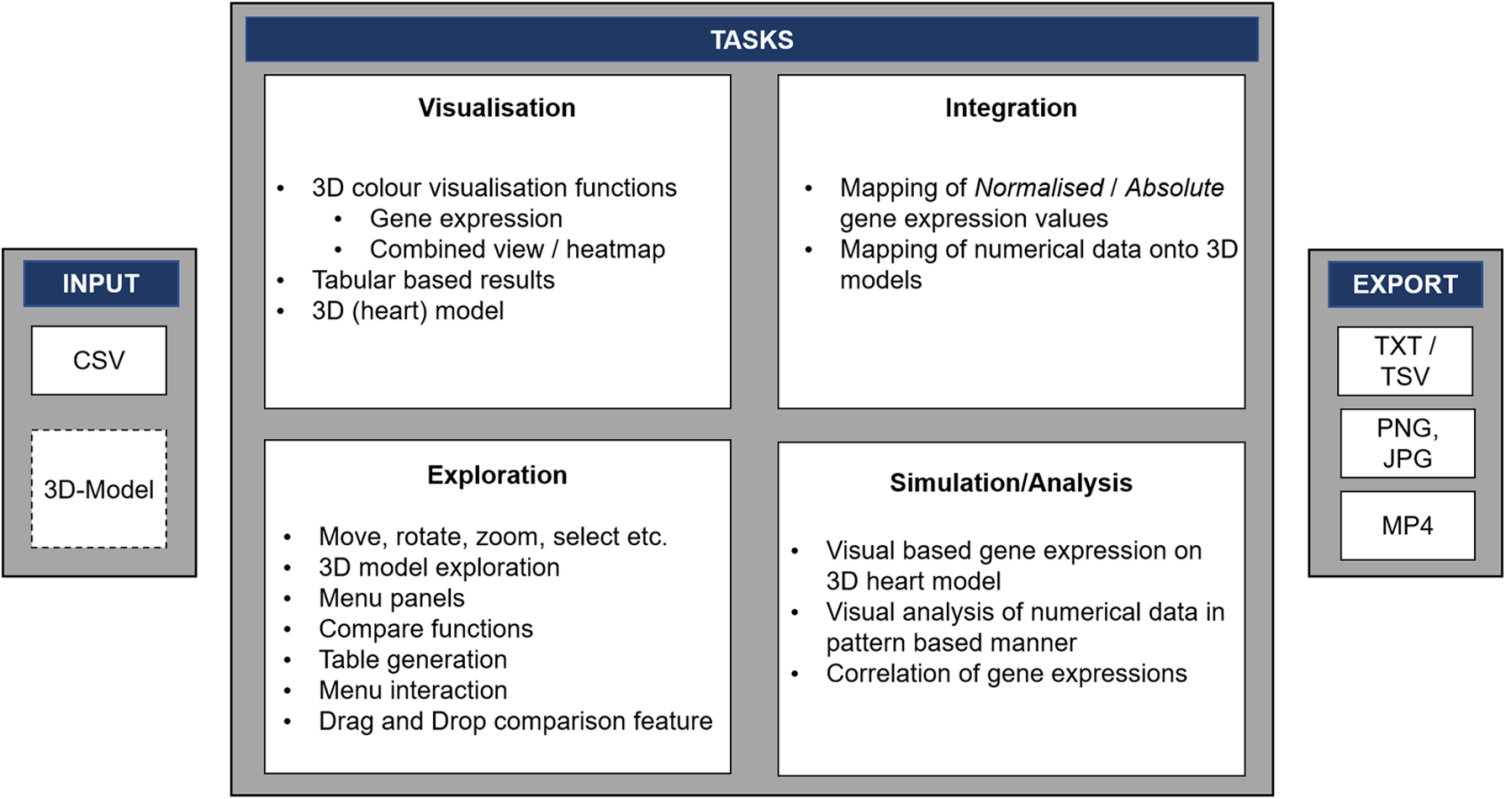
Functionalities of VR-Cardiomics

An overview of the functionalities of VR-Cardiomics is provided in Fig. 5. VR-Cardiomics is a framework prototype that can be used on a local machine with customized datasets and objects to meet the requirements.

Currently, datasets in CSV format are supported, although they need to be compatible with the number of pieces of the 3D object. For the 3D object, a format compatible with Unity is expected, which includes Filmbox (*fbx*), Digital Asset Exchange (*dae*) or Wavefront object (*obj*)^1^.

The environment offers a variety of export options for data. The data formats currently used are *TXT* with *TSV or CSV*-based outputs for the session logs and export functions of the tabular results. Screenshots can also be created within the environment in a *PNG* or *JPG* format, and a prototype function of screen recordings can be made in the *MP4* format.

### Use case

#### Dataset

The data used in this work were derived from the authors’ previous study [37], where the microdissection, tissue preparation, and RNA extraction protocols were detailed. In brief, adult mouse hearts were extracted and micro-dissected, and the resulting heart sections were prepared for RNA sequencing to obtain gene expression data. The 3D heart model was obtained using Amira software and virtually dissected using Maya software to produce a virtual 1:1 clone in the dissected heart sample. The raw gene expression data were normalized to log2 count-per-million (CPM) values using the EdgeR package, producing a data matrix with ∼75,000 transcripts and the 18 sections applied in this study. Visualization for VR-Cardiomics is based on log2 CPM normalized expression values of individual heart slices. These values were converted into a color code, producing a color gradient in the 3D heart model^2^.

#### Research questions

The following questions were investigated using the proposed tool:

I) Is there a region of interest (ROI) within a single gene expression pattern based on the 18 heart slices?
II) Is a certain gene expression value correlated with the expressions of other genes?
III) Which other genes show a high/low similarity in their gene expression pattern as the selected gene?
IV) Are there any visual ROIs in the gene expression patterns of two genes when compared next to each other?
V) Are there any ROIs based on the differences of two gene expressions patterns in any of the 18 slices?
VI) Are there any ROIs based on the grouping of individual sub-areas of the heart model?

#### Use of VR-Cardiomics to solve research questions

**Fig. 6:**
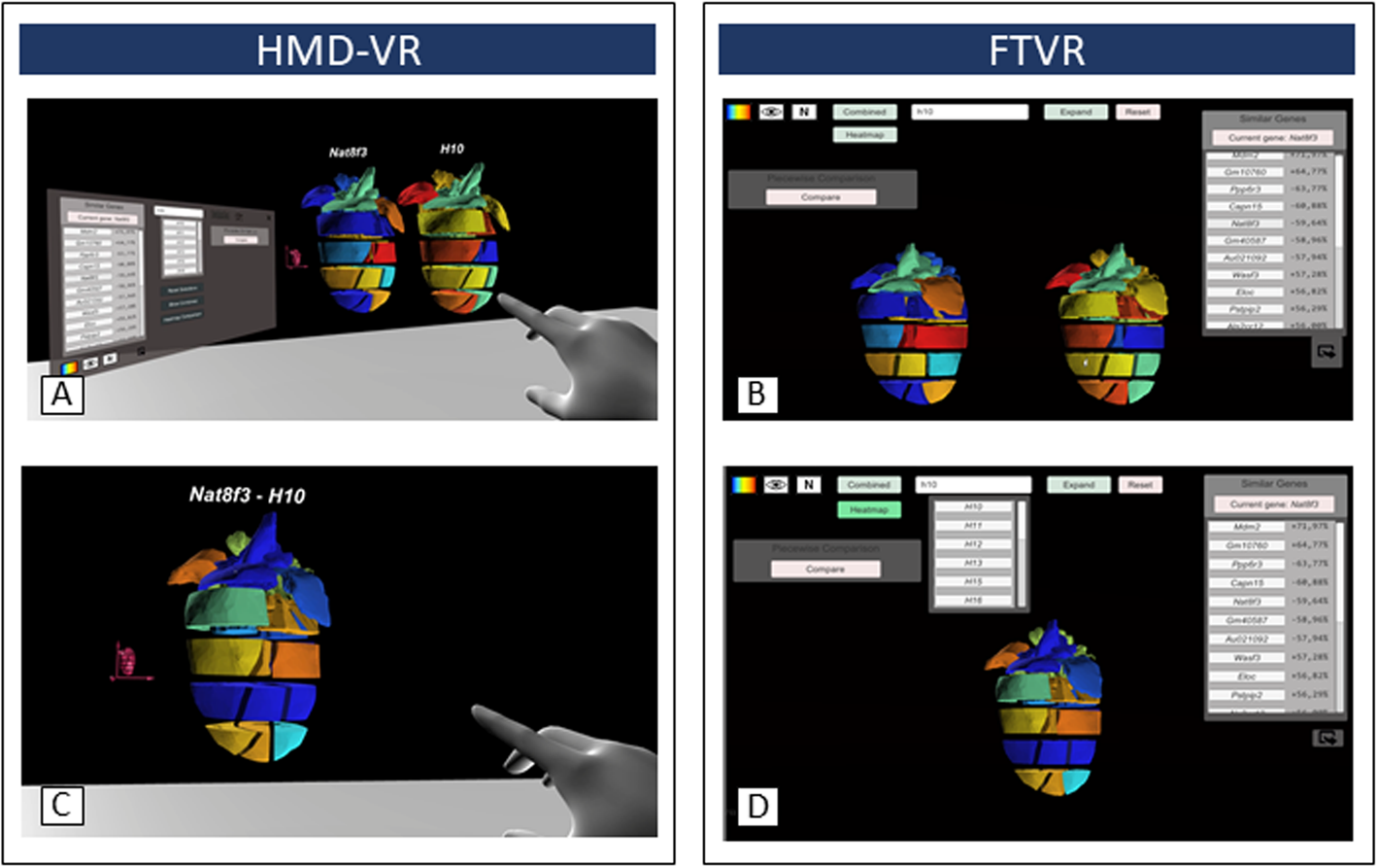
Comparison of both VR-Cardiomics prototypes for HMD-VR (left) and FTVR (right). Visual side-by-side comparison of two different gene expression patterns for A) HMD-VR and B) FTVR. *Heatmap comparison* in one model based on the differences of the gene expression values for C) HMD-VR and D) FTVR
II) During runtime, other gene expression patterns with similar distribution patterns are calculated and provided in tabular form. These are displayed as buttons and can thus be projected directly onto the model. Thus, correlations with the selected genes can be easily investigated.
III) The list of similar distribution patterns is calculated using absolute correlations with the original gene. They are calculated as a percentage of the normalized match and are listed in descending order of similarity.
IV) To determine the ROIs based on visual conspicuities to other gene expression values, additional models can be generated in the environment, and the information from additional gene patterns can be mapped onto them. Fig. 6 shows how a second heart model is generated next to the main model in the environment for a) the HMD-VR and b) FTVR. This allows the detection of abnormalities within specific regions.
V) However, if two or more gene expression values are to be examined solely based on their local differences in each of the 18 heart sections, a comparison of two gene patterns within one model can also be considered. This is shown in Fig. 6 for c) HMD-VR and d) FTVR. For each section, the difference is determined based on their normalized values, and this is again projected onto the model in a heat-map-like manner. High local differences are colored in red, and low local differences are shown in blue.
VI) In addition to looking at two different genes, ROIs can also be used to examine specific regions of the heart. To be able to target investigations based on sub-regions of the heart model, that is, certain groupings of the 18 sub-regions, two regions of the heart can be grouped using the *group selection*. Based on the expression of the selected gene, a list is calculated that matches the expressions specifically for the selected groupings.

VR-Cardiomics can support solving the above-mentioned questions as follows:

I) The ROIs of a gene can be quickly identified and investigated through visualization. As shown in Fig. 6, the underlying genetic data are mapped onto the model and colored from a low to high normalized occurrence within the subsection.

## Implementations in VR and FTVR

## Discussion

The heart presents not only a unique challenge because of its complex anatomical structure and function, but also a great opportunity for an immersive data visualization in its native 3D environment. The proposed VR-Cardiomics system is the first IE that allows biologists to observe and analyze gene expression data on three-dimensional heart objects and thereby study their intricate patterns. It is among the first applications allowing a variety of visual analyses as well as tabular comparison functions of spatial gene expression values.

Because any 2D projection of the spatial expression pattern contains unavoidable artifacts, it is intuitive for heart experts to observe the heart in its native 3D environment. Biologists, as well as cardiologists, can benefit from this advantage because of a better understanding of the data being viewed. Observing the same data on a 2D desktop environment could lead to a loss of information and a potentially incorrect interpretation based on the abstraction into 2D, as well as a reduced understanding of the data.

VR-Cardiomics was developed to enhance the interaction and understanding with the genetic data of the heart. Compared with an available 2D web application, the use of IEs offers numerous advantages. Although stereoscopic 3D is not physically 3D, it still supports many depth cues, unlike conventional 2D monitors. Particularly when using an HMD, the user experiences the advantage of a larger simulated space, i.e., the user is not limited to a fixed size of the given screen when viewing the data. In addition, although content projected onto a fixed 2D screen can be exchanged as compensation, the cost-benefit ratio is ultimately in favor of VR, owing to the higher level of physical navigation and the quicker emergence of an accompanying mental model. Thus, in VR-Cardiomics, the user is provided with a larger model for interaction and a 360^∘^ space for the arrangement of additional information and interfaces. This allows a much higher level of detail to be offered without compromising the clarity of the application because more space is available for information. This leads to the opportunity to allow the simultaneous use of multiple models with an HMD-VR without compromising the resolution or size in comparison to an FTVR.

Current consumer VR headsets are quite affordable, certainly by several orders of magnitude, compared to a more specialized display such as zSpace. Regarding visualization, one advantage of VR results from the superior lighting conditions of the HMD-VR, independent of the surroundings that are used^3^. Thus, while both devices have a comparable color saturation [45, 46], HMD-VR benefits from superior lightning conditions and thus offers an improved visual comparison for a color-coded design as implemented in VR-Cardiomics. Because it helps in gaining a better understanding of complex or three-dimensional data [35], VR is a well-known tool for various applications, e. g., for visualization in a radiology room [47]. However, immersion in a virtual world is also a common disadvantage of an HMD-VR, where the user is cut off from the physical environment. In certain cases, removal of the headset is nearly unavoidable, e. g., while exporting or transferring acquired data across software and/or systems. Depending on the type of tracking, the number of applications of an HMD-VR is considerably larger than that of an FTVR monitor.

By contrast, zSpace is a physical stereoscopic 3D monitor that allows for a considerably longer use during a given session. Furthermore, under a collaborative scenario, it is possible for collaborators to observe the projected 3D image by using additional polarization glasses without markers 4. For a 3D interaction, zSpace has only a single tethered stylus, which is more limited in the total number of action mappings and overall reach. However, owing to its form factor, interaction with the zSpace stylus can be supplemented using a keyboard and mouse input. Regarding the haptics, both types of VR devices have similar vibro-tactile feedback through their interaction mediums, controllers, and stylus.

Currently, a wide range of Unity-supported VR, AR, and FTVR devices are available that can be compatible with VR-Cardiomics. Each of these immersive devices carries a different cost versus quality trade-off, and thus having a variety of choices provides more flexibility for end-users. For example, the Rift S from the Oculus device used in this study is a low-cost model as of 2021. Although VR-Cardiomics is a framework for analyzing genetic data on the heart, its functions can be transferred to other domains. Therefore, there is no specific limitation to biologically related data, which means that the application can be useful in other areas (e. g., the brain) as well as for other types of data (e. g., metabolomic data).

Future development of the VR-Cardiomics system will be driven mainly by the needs of biologists’. Currently, one key area of focus is the incorporation of a single-cell resolution, thereby combining single-cell features as well as cell-to-cell interactions in a semi-native virtual environment of the tissue. Further, the incorporation of pathological features of the heart will be of significant utility to cardiologists. The incorporation of generic spatially resolved transcriptomics datasets without the need to first develop virtual 3D models will greatly increase the accessibility of the systems to biologists.

## Conclusion and outlook

In this paper, VR-Cardiomics, which to the best of the authors’ knowledge, is the first spatially resolved transcriptomics analysis application in VR, is presented. This application has been implemented and presented for use in VR through Oculus HMDs as well as for FTVR using zSpace. VR-Cardiomics is a novel interactive gene expression tool optimized for use in IE.

Currently, its capabilities are limited, however. Future improvements will focus on optimizing the input and output functions. This includes alternative export options in addition to the described text files, as well as an improved threshold regulation of the data. Furthermore, the authors would like to achieve a more generic implementation of VR-Cardiomics to make it available as a full framework application for other data domains. Based on experiences with different devices, the authors assume that in everyday use, the zSpace device will prevail in terms of integration into the work environment and its simplified use. In terms of the quality of the results, the customization of the environment, and the amount of simultaneously comparable data, the VR setup offers several advantages. However, user studies will be important for a final judgment on the suitability of both devices, and the authors have a plan to conduct such studies in the future.

The authors anticipate that VR-Cardiomics can be extended to accommodate a wide range of novel applications of spatially resolved transcriptomic data beyond the heart. With the advantage of spatial biology technologies converging to single-cell resolutions, VR-Cardiomics has the potential to visualize the whole transcriptome of individual cells, enabling unprecedented levels of details of spatial expression patterns [2, 4, 5]. The authors posit that VR-Cardiomics is a unique and valuable contribution to the fields of cardiac development, spatial biology, immersive analytics, and other areas.

## Supplementary Information

Additional file 1: Supplementary video.

## Data Availability

Applications and source codes are available on GitHub. The HMD-VR application is available at https://github.com/Ramialison-Lab/3DCardiomicsVR, and FTVR applications are available at https://github.com/Ramialison-Lab/3DCardiomicsZSpace.

## Abbreviations

EOL: End of Life
FTVR: Fish Tank Virtual Reality
HMD: Head Mounted Display
IA: Immersive Analytics
IE: Immersive Environment
ROI: Region of Interest
UI: User Interface
VR: Virtual Reality

## Footnotes

1 https://docs.unity3d.com/Manual/3D-formats.html

2 The genetic data used here were generated for the development process.

3 Tracking accuracy can still be affected.

4 The view is still tracked only for the operator
